# Incorporating modified team-based learning into a flipped basic medical laboratory course: impact on student performance and perceptions

**DOI:** 10.1186/s12909-022-03676-1

**Published:** 2022-08-06

**Authors:** Jing Shen, Hongyan Qi, Yingying Chen, Ruhuan Mei, Cencen Sun, Zhengyang Wang

**Affiliations:** 1 Department of Pathology and Pathophysiology, and Department of Medical Oncology of the Second Affiliated Hospital, Zhejiang University School of Medicine, Hangzhou, 310058 China; 2 Department of Pathology and Pathophysiology, Zhejiang University School of Medicine, Hangzhou, 310058 China; 3 Experimental Teaching Center of Basic Medicine, Zhejiang University School of Medicine, Hangzhou, 310058 China; 4 Department of Pulmonary and Critical Care Medicine, Sir Run Run Shaw Hospital, Zhejiang University School of Medicine, Hangzhou, 310016 China

**Keywords:** Flipped classroom, Team-based learning, Basic medical laboratory course, Medical education

## Abstract

**Background:**

Basic medical laboratory courses (BMLCs) play an essential role in medical education and offer several benefits to students. Although various student-centered and active learning strategies have been increasingly incorporated into medical education, their applications in BMLCs are limited. This paper aimed to explore the educational effects of a flipped classroom (FC) combined with team-based learning (TBL) strategy in BMLCs at Zhejiang University School of Medicine.

**Methods:**

Four hundred eight 3rd-Year medical students were assigned to either the FC-TBL group (*n* = 235) or the FC group (*n* = 173) to complete three experiments on the respiration block of BMLCs. The two groups’ immediate and long-term academic performance were compared, and the FC-TBL students’ perceptions of different instructional strategies were surveyed.

**Results:**

Students in the FC-TBL group scored higher on the immediate post-tests after class and higher on the final exams in two of the three experiment sessions. They preferred FC-TBL to FC for its higher engagement, more feedback, and better learning environment. Students felt the FC with TBL blended instructional strategy stimulated their interest in learning and deep thinking.

**Conclusions:**

Compared with the FC group, students in the FC-TBL group improved academic performance and had a more positive experience overall. Our findings support the feasibility and advantage of the flipped classroom with team-based learning as a blended learning strategy in the BMLC curriculum.

## Introduction

Laboratory instruction plays a critical role in science education. It is considered to be beneficial to students in many ways, such as promoting knowledge mastery, enhancing practical skills, developing scientific reasoning, and fostering teamwork [1]. However, several factors, including lack of inquiry-type activities and effective assessment, still restrain the achievement of these learning outcomes. With the global promotion of the outcome-based education (OBE) approach, basic medical laboratory courses (BMLCs) in China are undergoing significant variances. The traditional laboratory instruction based on confirmatory experiments is gradually shifting to more emphasis on knowledge application oriented by inquiry and innovation, thus supporting the cultivation of scientifically competent graduates [2, 3]. For example, the “cookbook” teaching in which students follow a predetermined set of directions is partially replaced by scientific question-driven laboratories, and virtual simulation experiments based on clinical scenarios are developed to facilitate authentic learning [3–5].

To maximize the learning effect, active learning strategies are supposed to be adopted in laboratory teaching to engage students in the whole learning process including experimental preparation and conduction as well. Although active learning activities have been shown to accelerate learning, one of the concerns is that they tend to require more in-class time and thus reduce time on content coverage [6]. The emergence of the flipped classroom (FC) appears to be an effective solution, as it provides an approach to introducing content as homework, allowing the class time to be used for active learning. In the FC, students learn foundational knowledge at their preferred time, place, and pace, and then perform high-level cognitive tasks or solve complex problems along with their colleagues and instructors inside the class [7]. This hybrid teaching format improves time efficiency and encourages students to become independent and self-responsible learners [8]. In 2016, Zhejiang University School of Medicine started to implement FC teaching in an integrated laboratory course consisting of basic medical experiments and independent novel research projects for 3rd-year medical students. Due to the high requirements of inquiry-based or problem-based instructional sets in class, students need to be well-prepared for and think deeply about the practical activity. However, a great challenge encountered in the course is the students’ insufficient experimental preparation and active thinking on scientific issues, which directly affects their learning performance. Other studies have reported similar problems with limited pre-class learning and in-class outcomes in FC [9].

Team-based learning (TBL) is a student-centered instructional strategy that promotes active learning through a sequence of procedures that includes individual work, teamwork, and immediate feedback [10]. There is growing evidence that TBL improves academic outcomes compared to traditional teaching strategies [11, 12]. TBL has also been reported to enhance student engagement, satisfaction, and collaboration [13, 14]. One of the key characteristics that make TBL effective is promoting students’ accountability. At first, students feel accountable because they want to improve their scores on individual tests, then they come to feel accountable to their teammates because they want to contribute to the success of their team. Therefore, TBL can effectively improve the efficiency and active learning outcome of flipped teaching.

The FC with TBL approach has been reported to change the way students learn, and enhance students’ knowledge, problem-solving ability, and learning satisfaction [15–17]. However, these studies are mainly focused on lecture courses and a few on laboratory instruction [18]. Since laboratory courses are typically set up to be more active in nature than a lecture course, it’s necessary to investigate whether the combination of active strategies can maximize learning and retention. In addition, most studies on FC and TBL are compared with traditional didactic lectures. It is still not clear whether FC combined with TBL has better academic outcomes and satisfaction than flipped teaching alone. Accordingly, the purpose of the present study was to compare student performance and perceptions of the FC with TBL blended learning strategy with that of the FC approach in a basic medical laboratory course.

## Methods

### Design and participants

The basic medical experiments of the integrated laboratory course in Zhejiang University School of Medicine include four practical blocks (neurology and skeletal muscle, circulation, respiration, and urology) run in a FC format for 9 weeks, with 2 to 3 experiments in each block. In 2020, we piloted three modified TBL sessions combined with flipped classroom (FC-TBL) in the respiration block, while the other blocks still used FC pedagogy. The three experiments covered in the respiration block were Fundamental Design of Respiratory Function Studies, Regulation of Respiration, and Virtual Patient Experiment of Respiratory Failure. A total of 408 3rd-Year medical students were assigned to either the FC-TBL group (*n* = 235) or the FC group (*n* = 173) in the respiration block by convenience sampling. A final examination requiring recall of facts and application of knowledge from all four blocks takes place at the end of this course. All of the procedures in this study were approved by the Ethics Committee of Zhejiang University School of Medicine, and informed consent was obtained from the participants.

### Structure of FC-TBL

While the learning outcomes remained the same, three experiments in the respiration block adopted the FC-TBL format and were designed to run once per week for 6 h each. Teams of five to six students were allocated by the researchers based on students’ gender and previous semester grades, so that each team had a diverse mix of students. Then five teams formed a class. These student teams and classes remained constant for the entire respiration block. The instructors for all FC-TBL classes were two content experts with TBL teaching experience, one of whom has been trained by the Team-Based Learning Collaborative (TBLC). FC-TBL methods were followed as outlined in Table 1. Before class, we provided online resources including pre-recorded lecture videos, Microsoft PowerPoint slides, and reading assignments. Students were required to learn the pre-class materials to prepare for scientific problems and experimental design. In class, students performed the individual readiness assurance test (IRAT) (15 min), consisting of 15 multiple choice questions, with one single best answer. Then it was followed by the same test as a team readiness assurance test (TRAT) (30 min) by using scratch cards. Teams simultaneously shared their answers, followed by an instructor-facilitated large group discussion (30 min). The instructor also offered clarification, particularly when teams encountered difficulties or disputes. After the readiness assurance tests and discussion, there was a short summary session held by students to further summarize the experimental design, procedure, and expected results. Students then moved on to the application activities (260 min): first conducted experiments in groups, then discussed five questions related to the experiment results in their teams according to the principles of 4S (significant problem, same problem, specific choice, and simultaneous reporting). At the end of the class, students were required to complete a post-test. After class, in addition to the experiment report, students were also asked to assign a score based on their teammates’ contribution to the team’s productivity for peer evaluation. However, the score of peer evaluation was not included in student grading.

**Table 1:** Structure of flipped classroom combined with team-based learning (FC-TBL) and flipped classroom (FC)

Component	FC-TBL	FC
Pre-class (1 ~ 2 h)	Students learn pre-recorded lectures and supplementary materials	Students learn pre-recorded lectures and prepare the presentation according to the questions provided by the teacher
In-class (6 h)	15 min: IRAT 30 min: TRAT 30 min: teams simultaneously share their answers, followed by large group discussion 15 min: students summarize key points 230 min: students do experiments in groups 30 min: teams analyze the experiment results, followed by a large group discussion 10 min: post-test	15 min: pre-test (same questions as IRAT) 60 min: students have presentations and answer questions 15 min: teacher summarizes key points 230 min: students do experiments in groups 30 min: students answer the teacher’s questions about the experiment results 10 min: post-test
Post-class (1 h)	Peer evaluation Students finish the experiment report	Students finish the experiment report

### Structure of FC

The same instructors as FC-TBL implemented the traditional flipped classroom teaching in FC (Table 1). Before the class, although the same online resources were allocated to students as in the FC-TBL group, students in the FC group were also asked to prepare presentations based on questions related to the experiment (basic knowledge, operations, and predicted results of the experiment). In class, students first took a pre-test which was consistent with the questions of IRAT in the FC-TBL group. Then, the instructor randomly selected one student from each experimental operation group to make presentations according to the questions prepared before class. Other students were encouraged to ask questions and have discussions with the presenters, while the instructor provide necessary clarification and made a summary of the key points at the end of the presentation. After that, students worked in small groups of three to complete the experiment, followed by a question-and-answer session. At the end of the class, students were required to complete the same post-test as the FC-TBL group.

### Data collection and analysis

Pre-test, post-test, and final exam scores of the three experiment sessions in the respiration block for all participants were collected and analyzed using the two-tailed Student’s *t* test with SPSS 26.0 program. After completion of the three sessions of the respiration block, all participating students in the FC-TBL group completed questionnaires assessing their experience with FC-TBL (respiration block) and FC (other blocks) as well as satisfaction and perceptions about engagement, teamwork, feedback, and the qualities of the learning environment. The questionnaires included 12 closed items using a five-point Likert scale (1 being ‘strongly disagree’, and 5 being ‘strongly agree’). The Cronbach’s *α* coefficient of the scale was 0.95. Open-ended questions were also utilized to assess strengths and areas for improvement. Quantitative results of closed items were analyzed using the two-tailed Student’s *t* test. Thematic analysis was performed to code and categorize qualitative data into themes [18]. *P* < 0.05 was considered statistically significant.

## Results

### Academic Performance

**Fig. 1:**
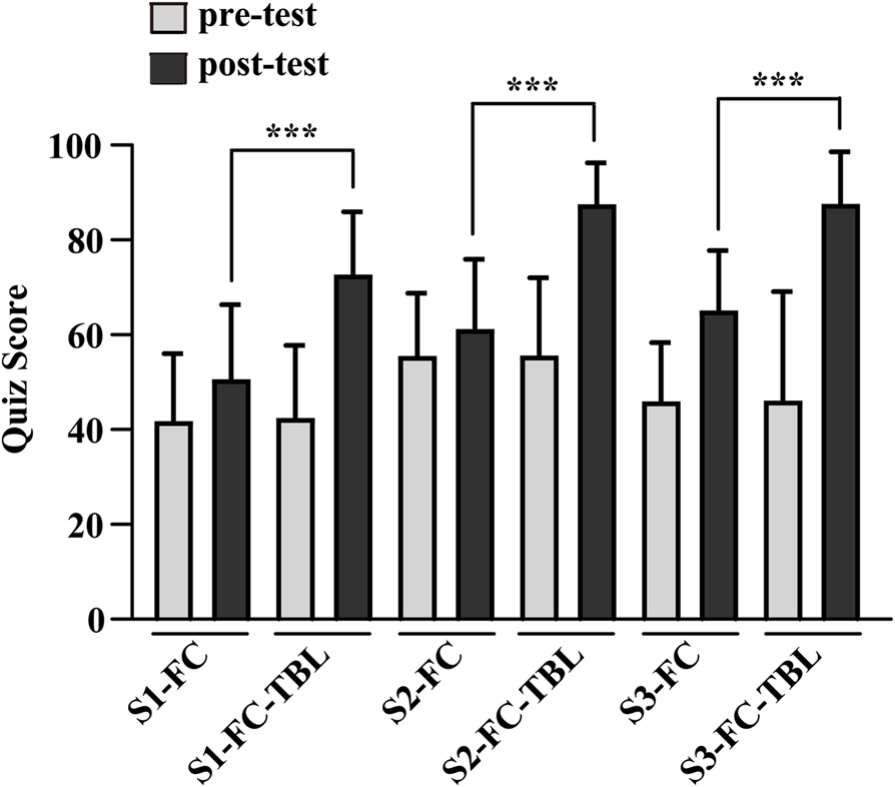
Comparison of pre-test and post-test scores between the FC and FC-TBL groups in three experiment sessions. Both FC and FC-TBL groups completed the same pre- and post-tests in each experiment session. Although no differences were found in pre-test scores between the two groups across all three sessions, the post-test scores were significantly higher in the FC-TBL group than in the FC group. S1, session 1; S2, session 2; S3, session 3. ***P < 0.001, compared with the post-test scores of the FC group in the same session

**Fig. 2:**
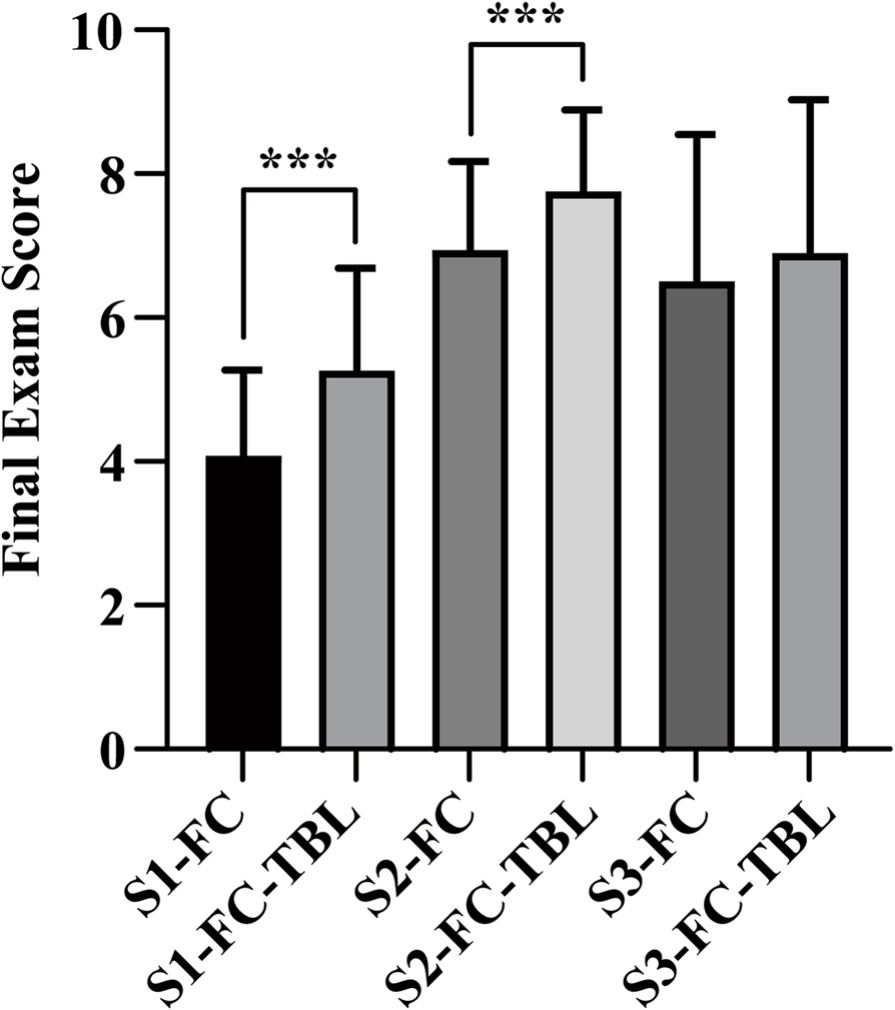
Comparison of final exam scores between the FC and FC-TBL groups in three experiment sessions. The FC-TBL group had higher final exam scores than the FC group in the first two sessions. ***P < 0.001, compared with the FC group in the same session

To evaluate students’ immediate academic performance, pre-test and post-test scores of the FC or FC-TBL groups were compared (Fig. 1). No differences were found in the pre-test scores between the two groups in all three sessions, indicating that students had similar levels of basic knowledge. However, the post-test scores of the FC-TBL group were significantly higher than that of the FC group. We also evaluated the final exam scores of both groups one month after the completion of four practical blocks of the course as long-term academic performance (Fig. 2). The FC-TBL group had higher final exam scores in the first two sessions than the FC group, while remaining the same in the third session.

### Students’ experience with FC and FC-TBL

In total, 231 out of 235 students (98.3% response rate) in the FC-TBL group completed the questionnaire regarding their experience with FC and FC-TBL. Table 2 shows student responses to closed items. Students reported higher individual and team engagement at FC-TBL than at FC. They received more feedback from peers and teachers in the FC-TBL class than FC did. In general, students were more satisfied with the organization and learning environment of FC-TBL than FC. Additionally, students perceived FC-TBL as superior to FC in the ability to stimulate their interest in learning, promote the practical application of knowledge, and improve their scientific reasoning and problem-solving skills.

**Table 2:** Comparison of students’ perceptions between FC-TBL and FC (*N* = 231)

Questions	FC-TBL (Mean ± SD)	FC (Mean ± SD)	*P* value^a^	Effect size
I actively participated in discussions and group activities	4.17 ± 0.71	3.62 ± 0.76	< 0.001	0.75
I kept up with the pace of the course	4.15 ± 0.71	3.59 ± 0.84	< 0.001	0.72
I made a great contribution to group activities	4.17 ± 0.66	3.95 ± 0.78	< 0.001	0.30
All team members made an effort to participate in group activities	4.07 ± 0.83	3.60 ± 0.91	< 0.001	0.54
All team members consistently paid attention during group activities	4.08 ± 0.79	3.56 ± 0.92	< 0.001	0.61
All team members made a great contribution to group activities	3.98 ± 0.80	3.65 ± 0.82	< 0.001	0.41
I received useful and timely feedback from team members	4.29 ± 0.65	3.50 ± 0.89	< 0.001	1.01
I received useful and timely feedback from the teacher	4.36 ± 0.64	3.93 ± 0.82	< 0.001	0.58
I am satisfied with the organization and learning environment of the course	4.26 ± 0.62	3.91 ± 0.78	< 0.001	0.50
The course has improved my interest in learning	4.10 ± 0.78	3.50 ± 0.87	< 0.001	0.73
The course has improved my ability to apply knowledge in practice	4.24 ± 0.63	3.67 ± 0.78	< 0.001	0.80
The course has improved my scientific reasoning and problem-solving skills	4.16 ± 0.68	3.67 ± 0.83	< 0.001	0.65

Likert Scale; 1 = Strongly Disagree, 2 = Disagree, 3 = Neither agree nor disagree, 4 = Agree, 5 = Strongly Agree

^a^Two groups are compared by the two-tailed Student’s *t* test and *P* < 0.05 is considered statistically significant

Responses to open-ended questions regarding students’ perceived best and most difficult features of FC-TBL and FC are shown in Tables 3 and 4 respectively. Students felt that the learning process of FC-TBL involves individual thinking and then teamwork, which effectively stimulated their interest in learning and inspired deep thinking. They also reported a high level of engagement and effective peer feedback in team-based activities and valued the discussions related to clinical practice. For FC, students found discussing according to the teacher’s guidance made the learning contents more comprehensive and logical, and also facilitated better pre-class preparation. There are some suggestions students made for FC-TBL, including enriching the content and type of questions, improving the efficiency of discussion, and increasing the relevance of pre-class learning materials to the in-class tests. Whereas for FC, students suggested improving engagement, reducing simple memorization of knowledge, and making the discussion more flexible.

**Table 3:** Students’ perceptions of FC-TBL, including best and most difficult features ( *N*= 231)

Theme	Examples of students’ comments	No. of similar responses
**Best features of FC-TBL**		
High level of engagement and effective peer feedback in group activities	*During the discussion and experiment, members of our team were highly engaged. Different opinions can be heard and efficiency was increased* *The group discussion was rich and fruitful. It helped us to better complete the experiment and explore its significance*	148/231
The learning process from individual test to group discussion stimulated interest in learning and inspired deep thinking	*Individual answers followed by group discussions work well. I learned a lot from the group discussion and thought more deeply* *I think it's great to think on your own first and then discuss with teammates. The opinions and feedback from others have deepened my understanding of knowledge*	124/231
More closely related to clinical practice	*The group discussion questions involving clinical cases are interesting and make the experimental results more relevant to our future practice*	50/231
**Most difficult features of FC-TBL**		
Students suggested that the content of the questions should be more comprehensive and have more types	*Hope to have more discussion questions related to experimental operations and statistical results* *There are only multiple choice questions and hope to have more types of questions*	68/231
Students suggested reducing the number of team members and improving the efficiency of the discussion	*Hope to reduce the size of the team to 3–5 people, and improve the efficiency of the discussion*	60/231
Students suggested the pre-readings should be more relevant to the test	*More focused and test-related pre-readings, such as a summary of basic knowledge, and a reminder of experimental operations*	13/231

**Table 4:** Students’ perceptions of FC, including best and most difficult features (*N* = 231)

Theme	Examples of students’ comments	No. of similar responses
**Best features of FC**		
The content of the discussion was more comprehensive and logical	*Students prepared the presentation as required by the teacher, covering all aspects of content from basic knowledge, and experimental operations to experiment results. The discussion was more comprehensive and logical*	82/231
Pre-class learning requirements were clearer and students were better prepared	*The teacher gave clear questions and presentation requirements before class, and most of the students were well prepared*	43/231
Group members undertook different pre-class preparation work with less pressure	*Before class, students in the experiment operation group can choose to prepare different presentation contents, which reduces the learning pressure*	43/231
**Most difficult features of FC**		
The engagement of students in the presentation and discussion sessions was relatively low	*When one student was presenting, other students were preparing their own presentations, and the learning effect was not good* *Students rarely asked questions and no real group discussions occurred*	85/231
The preparation task before class was mostly memorization, which was not very helpful to the experiment	*I felt like I spent a lot of time memorizing knowledge before class but still could not apply it well in the experiment*	59/231
The form of presentation and discussion is inflexible and cannot effectively stimulate interest in learning	*At the presentation and discussion session, I spent most of the time listening to other students or teachers. A little boring, and sometimes I couldn't concentrate* *The presentation and discussion session should be more flexible, allowing students to discuss freely*	40/231

## Discussion

Student-centered active learning strategies, including flipped teaching and learning in small groups, have been increasingly incorporated into healthcare education [17]. Due to the limited studies on these strategies in courses besides didactic lectures, it’s necessary to expand the range of their application in instruction, such as in laboratory courses. Therefore, in this study, we sought to explore the educational effects of a FC with TBL blended learning strategy in a Year 3 basic medical laboratory course. Our findings indicate that compared with the FC group, the students in the FC-TBL group had improved academic performance and an overall more positive experience.

Medical laboratory courses in China, such as BMLCs, usually consist of two parts: the first is teacher-directed lectures covering scientific concepts and experimental procedures, and then followed by student independent experiments [3, 4]. We switched the first part to flipped teaching in 2016, and further piloted three modified TBL sessions combined with FC in the respiration block in 2020. Although there have been series of evidence that TBL helped students learn better than traditional lectures [11, 12] [17], how it compares with flipped teaching remains unclear. An earlier study found that flipped teaching needs to be combined with cooperative learning to produce positive results in performance [19]. Interestingly, our findings also showed that the immediate learning outcomes of introducing TBL, an effective small group learning approach, in FC were overwhelmingly superior to those of flipped teaching that mainly focused on individual learning. There was a limited amount of peer interaction during class time in the FC alone group, with each student mainly getting help and feedback from the instructor, rather than from teammates. In contrast, the TBL approach of the FC-TBL group provides a series of group activities for the entire learning process including experimental preparation, operation, and analysis of results, allowing students to learn in a more cooperative environment. For the long-term learning performance, we examined the experimental design and implementation knowledge, as well as a mock study analysis in the final exam. Students in the FC-TBL group scored higher on final exams in two of the three experiment sessions than the FC group. It is considered that the sequential phases and core elements of TBL encourage the reconsolidation of knowledge which is vital to long-term knowledge retention and transfer [20, 21]. Our results, therefore, indicated that the interactive and application-oriented elements of TBL could also promote students’ knowledge retention and critical thinking in laboratory instruction.

To thoroughly understand students’ views towards the newly introduced blended learning approach in our laboratory course, we further compared their perceptions of FC and FC-TBL. Students preferred FC-TBL for its higher engagement and motivation, more feedback from peers and teachers, and better organization and learning environment. Students believed that both individuals and team members actively participated in and contributed to group activities in TBL classes (Table 2). They felt that *“during the discussion and experiment, members of the team were highly engaged”* and *“efficiency was increased”* (Table 3). As the application of knowledge and problem solving is the core of TBL class, group activities should always be closely related to it to improve students’ intrinsic motivation for learning [17]. Laboratory courses allow for the application of knowledge, skills, and reasoning, which could further increase the learning effectiveness of TBL application activities combined with it [22, 23]. In the two application activities of our laboratory class, FC-TBL group students first solved complex scientific problems through experiments and then discussed the results in groups, which made them feel more engaged and motivated. Moreover, providing clinical context in medical education can also help students better understand the content and improve students’ learning motivation [24]. Students felt that the provision of clinical scenarios for discussion during TBL made *“the experimental results more relevant to future practice”*, thus increasing their engagement in learning.

Another key feature favored by students in FC-TBL was the timely and helpful feedback, especially from team members (Table 2). Students also described that *“different opinions can be heard”* and *“the group discussion was rich and fruitful”* (Table 3). It has been demonstrated that immediate feedback can enhance students’ understanding of important concepts and is critical to knowledge acquisition, application, and retention [25, 26]. In TBL, a feedback-rich learning experience is designed based on peer interaction and instructor facilitation. Therefore, students will not be left in doubt regarding their understanding of the content since the feedback has been received through the readiness assurance process and during application activities. Meanwhile, students described their experience in FC as they *“rarely asked questions”* and *“no real group discussions occurred”* (Table 4). Insufficient engagement could impair students’ learning feedback. In FC class, both the discussion in the preparation stage of the experiment and the discussion on the results after completing the experiment were based on having students work on different problems, lacking challenges or contradictory conclusions in the same way, thus could greatly weaken the engagement and investment of students.

In addition, students rated FC-TBL higher than FC in improving learning interest and scientific reasoning skills (Table 2). Students found that the formal testing procedure, with the sequence of the readiness assurance process of TBL, stimulated their interest in learning and inspired deep thinking. They reported that *“individual answers followed by group discussions work well”*, and they *“learned a lot from the group discussion and thought more deeply”* (Table 3). In contrast, in the FC, students felt that they *“spent a lot of time memorizing knowledge before class but still could not apply it well in the experiment”* (Table 4). During TBL, students build on their learning by comparing their answers to other team members and participating in discussions to reach a consensus. In this way, the TBL format provides students with the opportunity to develop teamwork abilities and critical thinking skills, which are also the main learning outcomes that laboratory courses in medical education are intended to achieve [1, 17, 27].

For the best features of FC, students commented that *“preparing the presentation as required by the teacher”* made the knowledge learned *“more comprehensive and logical”* (Table 4). They also suggested that FC-TBL instruction could be improved by covering more knowledge contents, such as *“experimental operations and statistical results”* (Table 3). It is now agreed that while active learning emphasizes the shift from teaching to learning, teachers still need to support learning by providing necessary guidance [28]. In the sequential process of TBL, the instructor usually gives clarification or mini-lecture at the end of the readiness assurance tests to help the students to be prepared for solving complex problems [29]. For the laboratory class, according to the suggestions of students, in addition to improving the discussion questions, a student-directed summary step can be further included after the experiment is completed, such as drawing mind maps or finding connections between key knowledge. Other recommendations, including improving the efficiency of discussion and the relevance of pre-class learning materials to the tests, should also be included in future course improvements.

### Limitations

Although our study showed positive results favoring the FC with TBL blended learning, the study had some limitations. Three TBL sessions covering one block were piloted with the blended teaching, thus may not reflect the overall situation of the course. Further studies should be conducted to incorporate the new teaching format in the whole course to better assess its effectiveness. In addition, other learning outcomes outside testing that TBL may also promote have not been measured, such as the ability to work in a team, communicate, and solve problems. It is also possible that students gave more positive responses to the new teaching method simply because of the bias inherent in studying any new method of education. Therefore, longitudinal studies are needed to further understand its long-term impact on learning.

## Conclusion

This study found that the FC with TBL blended learning model improved students’ academic performance, including immediate and long-term learning outcomes, in a flipped basic medical laboratory course. Students exhibited a preference for FC-TBL over FC teaching for its higher engagement and motivation, more feedback from peers and teachers, and better organization and learning environment. Furthermore, this blended instructional strategy stimulated students’ interest in learning and inspired their deep thinking. However, as suggested by students, some advantages of traditional FC teaching, such as comprehensive discussion contents and clear pre-class learning directions, can be used to further optimize the FC-TBL approach.

## Data Availability

Datasets supporting the conclusions of this study are included within the article. Additional data at the level of individual students is not publicly available because of the potential for compromising student privacy but is available from the corresponding author on reasonable request.

## Abbreviations

BMLCs: Basic medical laboratory courses
FC: Flipped classroom
TBL: Team-based learning
IRAT: Individual readiness assurance test
TRAT: Team readiness assurance test
